# Bacterial and Fungal Community Responses to Long-Term Salinity Gradients in Natural Soils of Kazakhstan

**DOI:** 10.3390/microorganisms14061337

**Published:** 2026-06-14

**Authors:** Ainash Nauanova, Aisulu Onggarbay, Anel Ordabayeva, Bolat Abdigulov, Akgul Kassipkhan, Gulzhanat Maxutbekova, Aiman Nazarova, Alexandr Shevtsov

**Affiliations:** 1 Institute of Agriculture and Forestry, JSC «S. Seifullin Kazakh AgroTechnical University», Astana 010000, Kazakhstan; a.nauanova@kazatu.edu.kz (A.N.); aisulubauirzhan00@gmail.com (A.O.); a.kasipkhan@kazatu.edu.kz (A.K.); gulia_80-80@mail.ru (G.M.); a.nazarova@kazatu.edu.kz (A.N.); 2 “BIO-KATU” LLP, Astana 010000, Kazakhstan; 3 Center of Life Sciences, National Laboratory Astana, Nazarbayev University, Astana 010000, Kazakhstan; anel.ordabayeva@nu.edu.kz; 4 LLP “National Center of Biotechnology”, Astana 010000, Kazakhstan; abdigulovbolat3@gmail.com

**Keywords:** soil salinization, Kazakhstan, 16S rRNA gene, microbial diversity, Actinomycetota, Bacteroidota, Acidobacteriota, core microbiome

## Abstract

Natural saline–alkaline soils are widespread in Central Asia, yet microbial responses to salinity gradients and ionic composition remain poorly resolved. We profiled bacterial communities (16S rRNA V3–V4, Illumina MiSeq) in 20 topsoil (0–20 cm) samples from four regions of Kazakhstan spanning non-saline to highly saline conditions. Soil chemistry included pH, total mineralization (dry residue), and major ions (Na^+^, Cl^−^, SO_4_^2−^, HCO_3_^−^, Ca^2+^, Mg^2+^, K^+^). Alpha (Chao1, Shannon, observed ASVs) and beta diversity (Bray–Curtis; ANOSIM; PCoA) were evaluated across salinity classes. Soils were alkaline (pH 7.91–10.47) and covered a broad salinity range (256–26,312 mg/L), driven mainly by Na^+^ with chloride and/or sulfate. Alpha diversity remained stable across salinity classes, though dispersion increased under high salinity. Community composition differed significantly among classes (ANOSIM R = 0.428, *p* = 0.005), with partial PCoA separation and overlap, indicating gradual turnover along the salinity gradient. In contrast, fungal communities showed no significant response to salinity, with stable alpha and beta diversity across all samples and consistent dominance of *Ascomycota*. Communities were dominated by Actinomycetota (formerly Actinobacteriota), Bacteroidota, and Pseudomonadota (formerly Proteobacteria). Bacteroidota increased in highly saline soils (FDR q = 0.036), whereas Acidobacteriota decreased (FDR q = 0.052). Thermodesulfobacteriota (formerly Desulfobacterota) correlated positively with sulfate, and Cyanobacteriota negatively with chloride. Overall, Kazakhstan’s saline–alkaline soils show stable bacterial alpha diversity but moderate, ion-linked compositional shifts with enrichment of halotolerant taxa.

## 1. Introduction

Soil salinization is an excessive accumulation of soluble salts to levels that have a negative impact on crop productivity and the functioning of terrestrial ecosystems [1]. This process is one of the most common forms of land degradation in arid and semiarid regions of the world and is considered as one of the key factors in reducing the sustainability and productivity of agricultural landscapes [2]. Saline and brackish soils are found on all continents and are distributed in more than 100 countries around the world [3].

According to the latest global assessment by the Food and Agriculture Organization of the United Nations (FAO), the total area of soils affected by salinity and/or sodicity (salt-affected soils) is about 1.38 billion hectares, which corresponds to approximately 11% of the land area. In addition, about 1.0 billion hectares are classified as areas with a potential risk of salinization, characterized by moderate values of electrical conductivity of the soil solution (EC 0.75–2 dS/m), at which early changes in soil processes may already occur [4]. The most significant areas of saline soils are concentrated in regions with continental and arid climates, including Northern, Eastern, and Southern Africa, the Middle East, Central and Western China, the western United States, Central Asia, and Australia [5,6].

Global forecasts of climate change indicate a possible intensification of soil salinization processes in a number of arid regions by the end of the 21st century. According to model estimates for the period 2071–2100 compared with 1961–1990 under the RCP 8.5 scenario, the relative increase in average soil salinity in some countries may reach ≈15%, while in other regions a more moderate increase is predicted (about 3% in Australia). At the same time, for a number of territories, including the northwestern United States, Eastern Europe, Turkmenistan and western Kazakhstan, a tendency towards partial desalination of soils is expected due to changes in precipitation and evaporation patterns [7]. For the Central Asian regions, there is a high spatial heterogeneity and limited consistency of forecast estimates [8,9].

Kazakhstan is the largest country in Central Asia with a total area of about 2.7 million km^2^, which causes significant spatial heterogeneity of climatic conditions, water regime and soil cover, including salinization processes [10]. It is estimated that about 60% of the country’s territory is under threat of land degradation, with soil salinization being one of its key factors. About 41% of the territory of Kazakhstan is represented by saline, sodic, and solonchak soils, which indicates the scale and systemic nature of this problem [11]. The saline lands of the country are confined to various soil–halo–geochemical provinces, differing in the origin of salts, their chemical composition and migration mechanisms. Within the framework of soil–halo–geochemical zoning, four main salt accumulation provinces are distinguished in Kazakhstan: the Caspian (sulfate–chloride and chloride types of salinity), the Aral (chloride–sulfate type), the Balkhash (soda–sulfate type) and the Kara Sea basin (chloride–sulfate type) [12].

Despite significant progress in the study of the physico-chemical properties of saline soils in Kazakhstan, the biological aspects of the functioning of salt marsh ecosystems remain studied to a much lesser extent. In particular, information on the structure, diversity, and ecological role of soil microbial communities under conditions of various types and intensities of salinity remains limited. Meanwhile, soil microorganisms are a key component of soil ecosystems, determining the intensity of transformation of organic matter, the biogeochemical cycle of nutrients and the formation of functional stability of soils [13,14,15]. Under salinization conditions, microbial communities play a central role in soil adaptation to osmotic and ionic stress, which is directly related to the preservation of their productive and ecological potential [16,17].

This paper analyzes the taxonomic structure and diversity of the microbiome of soils in Kazakhstan with different levels of salinity. Most previous studies have focused on agricultural or experimentally salinized soils, whereas information on microbial communities in long-term naturally formed saline–alkaline soils of Central Asia remains limited. Recent high-throughput sequencing studies have highlighted the importance of specific ions and salinity gradients in shaping microbial community assembly; however, such patterns remain poorly characterized in natural steppe ecosystems of Kazakhstan.

The present study addresses this gap by analyzing bacterial community structure across a broad natural environmental (chemical) gradient of salinity and ionic composition in saline–alkaline soils of Kazakhstan. We aimed to (i) characterize taxonomic shifts along the salinity gradient, (ii) evaluate the relative influence of individual ions on community composition, and (iii) assess the ecological significance of these patterns for understanding microbial adaptation to extreme environments. In addition, we explored fungal community patterns to assess whether similar salinity-driven trends are observed across prokaryotes and unicellular eukaryotes.

## 2. Materials and Methods

### 2.1. Study Area and Sampling

Soil samples were collected in four regions of Kazakhstan—Pavlodar, Akmola, Karaganda, and Atyrau—representing different soil–halo–geochemical salt accumulation provinces sensu Issanova et al. [12]. Sampling sites in the Atyrau region are located within the sulfate–chloride (chloride-dominated) salt accumulation province of the Caspian Sea drainage basin, which is characterized by long-term accumulation of predominantly sodium chlorides associated with marine sediments, groundwater discharge, and aeolian salt transport. In contrast, soils from the Pavlodar, Akmola, and Karaganda regions belong to subbasins of the chloride–sulfate salt accumulation province of the Kara Sea drainage basin, where salinization processes are controlled by continental runoff, steppe–dry steppe climatic conditions, and large-scale geochemical transit and partial accumulation of salts [12].

The studied soils belong mainly to the steppe and arid soil ecosystems of Northern, Central and Western Kazakhstan and are represented by various taxonomic groups according to the international classification WRB (World Reference Base for Soil Resources). In the northern and central regions (Pavlodar, Akmola and Karaganda regions), chestnut and light chestnut soils predominate, represented mainly by Haplic Kastanozems, Calcic Kastanozems and Solonetz. These soils are formed in the conditions of the continental climate of dry steppes and are characterized by carbonate, alkaline reaction of the medium and periodic accumulation of easily soluble salts.

In western Kazakhstan (Atyrau region), arid soils of the semi-desert zone are widespread, including Solonchaks and Solonetz, which are formed under the influence of highly mineralized groundwater and evaporative water regime. These soils are characterized by the accumulation of sodium chloride and sulfate salts, high alkalinity and pronounced salinity of the soil profile [18].

Soil type is one of the key factors determining the structure and functional diversity of soil microbial communities, as differences in mineralogical composition, texture, organic matter, and water–salt regime significantly affect the formation of the soil microbiome [19,20,21].

**Figure 1:**
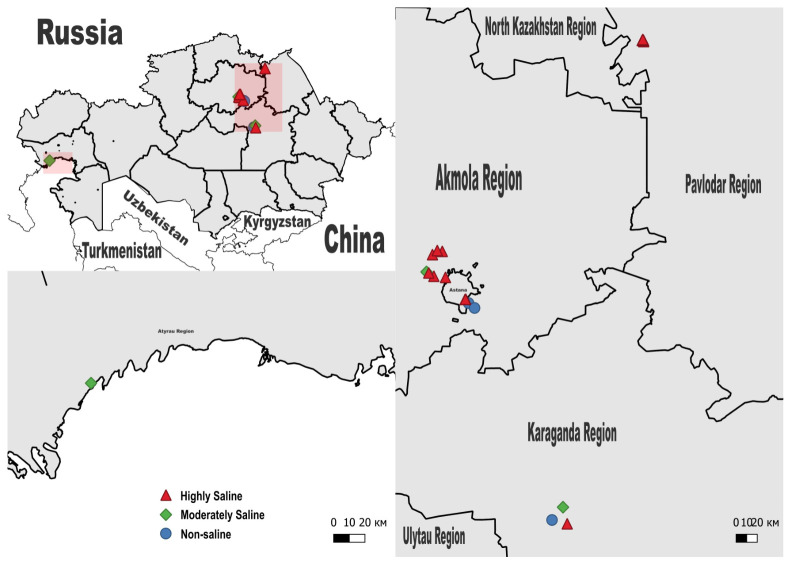
Geographic location of the soil sampling sites in Kazakhstan. A total of 20 sites were sampled across different regions of the country. [Source: The map was generated using QGIS version 3.44.7 (https://www.qgis.org)].

All study areas are characterized by a sharply continental climate; however, they differ markedly in thermal and moisture regimes. According to official meteorological records, the northern and central regions (Pavlodar, Akmola, and Karaganda) are characterized by relatively low mean annual air temperatures (approximately 4.7–5.4 °C), cold winters, moderate summer warming, and moderate annual precipitation (approximately 296–353 mm). In these regions, mean annual relative humidity ranges from 64.3% to 72.7%, and average wind speeds vary between 3.3 and 4.5 m/s. The Atyrau region, located in the arid zone of western Kazakhstan, exhibits a warmer and drier climatic regime, with a higher mean annual air temperature (approximately 12.2 °C), hot summers, milder winters, lower annual precipitation (about 205 mm), and reduced relative humidity (around 58.6%) (Table S1). Soil sampling was conducted at 20 sites (Figure 1, Table S2).

The geographical coordinates of all sampling points were determined using a GPS receiver (Garmin Montana 700, Garmin, Olathe, KS, USA) and are shown in Figure 1. The spatial location of the studied sites is shown on a map (Figure 1) constructed using the QGIS software. The coordinates of the points ranged from 46 to 52° N, 48–76° E, covering the main natural zones of Kazakhstan—from dry steppes to semi—deserts.

The cartographic representation of sampling sites makes it possible to take into account for spatial heterogeneity of soil conditions and is widely used in studies of the soil microbiome [22,23].

At each site, a 10 × 10 m plot was established, within which 16 individual soil subsamples were collected. Sampling was performed in areas free of visible vegetation; the minimum distance to the nearest plant was at least 20 cm in order to minimize rhizosphere effects.

Soil sampling was carried out from the surface humus horizon (A horizon) corresponding to the arable or turf layer of the soil.

This horizon is characterized by maximum biological activity, increased organic matter content, and the highest density of soil microorganisms. In the steppe soils of Kazakhstan, the thickness of the humus horizon is usually in the range of 15–30 cm, which corresponds to the selected sampling depth on average [18].

During sampling, special attention was paid to the distribution areas of intrazonal soils formed under the influence of salinization processes, which is typical for arid and semi-arid landscapes of the steppe zone of Kazakhstan. Such soils are formed in conditions of close occurrence of mineralized groundwater, poor drainage and intense evaporation, which leads to the accumulation of slightly soluble salts in the upper horizons of the soil profile. The presence of salinization processes has a significant impact on the physico-chemical properties of soils, including the ionic composition of the soil solution, osmotic potential and nutrient availability. The use of the upper humus horizon is a standard practice in studies of the soil microbiome, since it is this layer that determines the basic biogeochemical processes of the soil, including the mineralization of organic matter and the transformation of nutrients [24,25]. In addition, in conditions of saline and brackish soils, it is the upper horizon that is most sensitive to changes in the salt regime, which affects the structure and functional diversity of microbial communities.

Each subsample was placed into a separate sterile plastic bag and transported to the laboratory at 4–8 °C within 24 h after collection. Under laboratory conditions, the 16 subsamples obtained from each plot were thoroughly homogenized to form a composite sample, which was subsequently divided into two equal portions. One portion was used for the determination of soil physicochemical properties, while the second portion was sieved through a 2 mm mesh and stored at −80 °C until DNA extraction.

### 2.2. Determination of Soil Physicochemical Properties

All chemical analyses were performed on air-dried soil samples sieved to <2 mm. Soil organic carbon (SOC) was determined by high-temperature dry combustion using an elemental analyzer (vario MAX cube CNS, Elementar Analysensysteme GmbH, Langenselbold, Germany) in accordance with ISO 10694 [26]. The measured SOC values were converted to humus content using the van Bemmelen conversion factor (1.724), which is widely applied in soil organic matter assessments [27]. Nitrate nitrogen (NO_3_^−^–N) was extracted with a 1% potassium aluminum sulfate solution at a soil-to-extractant ratio of 1:2.5 and quantified potentiometrically using a nitrate-selective electrode, following established standard procedures for mineral nitrogen determination in soils [28]. Available phosphorus was determined using the Olsen bicarbonate extraction method (0.5 M NaHCO_3_, pH 8.5), which is specifically recommended for neutral to calcareous soils [29]. The alkaline extractant suppresses Ca^2+^ activity and promotes the release of calcium-bound phosphate forms, ensuring reliable estimation of plant-available phosphorus [30]. Phosphorus concentration in the extracts was measured spectrophotometrically using the molybdenum blue method on a UV–Vis spectrophotometer (Photolab VIS 7100, WTW, Xylem, Weilheim, Germany) at a wavelength of 882 nm, following standard colorimetric procedures. Exchangeable potassium was extracted with 1 M ammonium acetate solution (pH 7.0) via cation exchange reactions, whereby NH_4_^+^ ions displace K^+^ from soil exchange sites. Potassium concentration in the extract was determined by flame photometry using a digital flame photometer (BWB-XP, BWB Technologies, Newbury, UK) at a wavelength of 766 nm, in accordance with ISO/TS 22171:2023 [31]. Soil reaction (pH in water) was measured potentiometrically in a 1:5 (*w*/*v*) soil-to-deionized water suspension in accordance with ISO 10390:2021 [32]. Water-soluble cations (Ca^2+^, Mg^2+^, Na^+^, K^+^) and anions (Cl^−^, SO_4_^2−^) were determined from 1:5 soil water extracts using capillary electrophoresis on a capillary electrophoresis system (Kapel-105M, Lumex, St. Petersburg, Russia). The method is based on ion separation according to electrophoretic mobility in a fused silica capillary under an applied electric field and allows high-resolution characterization of soil salinity composition [8]. Carbonate (CO_3_^2−^) and bicarbonate (HCO_3_^−^) alkalinity in soil water extracts was determined by two-endpoint potentiometric titration with 0.02 M sulfuric acid, corresponding to phenolphthalein and methyl orange equivalence points, respectively, following internationally accepted analytical protocols [28,33]. Total dissolved solids (TDS), expressed as dry residue, were determined gravimetrically by evaporating a known volume of filtered soil water extract and drying the residue to constant mass at 103–105 °C, in accordance with Standard Methods 2540 C [33].

### 2.3. DNA Extraction from Soil, PCR Amplification and Sequencing on the Illumina MiSeq Platform

Total DNA was isolated from 5 g of soil according to the protocol proposed by Biver et al. [34]. Libraries for soil microbiocenosis analysis were prepared in accordance with the Illumina 16S Metagenomic Sequencing Library Preparation protocol [35]. The V3–V4 regions of the 16S rRNA gene were amplified using universal primers described by Klindworth et al. [36], with Illumina overhang adapters: Forward: 5′-TCGTCGGCAGCGTCAGATGTGTATAAGAGACAGCCTACGGGNGGCWGCAG-3′; Reverse: 5′-GTCTCGTGGGCTCGGAGATGTGTATAAGAGACAGGACTACHVGGGTATCTAATCC-3′. For fungal community analysis, the ITS1 region was amplified using Illumina adapter-linked primers NGS_ITS1-30 and NGS_ITS1-217R, based on previously described ITS1 primer systems [37]. The primer sequences were as follows: NGS_ITS1-30 (5′-TCGTCGGCAGCGTCAGATGTGTATAAGAGACAGGTCCCTGCCCTTTGTACACA-3′) and NGS_ITS1-217R (5′-GTCTCGTGGGCTCGGAGATGTGTATAAGAGACAGTTTCGCTGCGTTCTTCATCG-3′). PCR amplification was performed in a volume of 25 µL using Phusion^®^ High-Fidelity DNA polymerase with HF Buffer (Thermo Scientific Baltics UAB, Vilnius, Lithuania). The resulting amplicons were purified using AMPure XP magnetic particles (Beckman Coulter, Beverly, MA, USA), after which the libraries were indexed using the Nextera XT Index Kit (Illumina, San Diego, CA, USA). The prepared libraries were normalized, pooled, and sequenced on the Illumina MiSeq platform using the MiSeq Reagent Kit v3 (2 × 300 bp).

### 2.4. Bioinformatics and Statistical Analysis

For 16S rRNA gene amplicon data, raw paired-end sequencing data were quality-filtered using Sickle v1.33 [38] with a minimum quality score of 20, applying the default minimum read length setting. Quality-filtered sequences were processed using QIIME 2 version 2024.5 [39]. Denoising was performed with DADA2 [40], including primer trimming of 17 bp from forward reads and 21 bp from reverse reads, to generate amplicon sequence variants (ASVs). Taxonomic classification was assigned using a pre-trained Naive Bayes classifier based on the SILVA 138 reference database, using a 99% similarity threshold [41,42]. Fungal taxonomic classification was performed using the UNITE v10.0 reference database, trained for use with QIIME 2 [43].

For *ITS* amplicon data targeting the fungal community, raw paired-end reads were quality-filtered using QIIME 2 version 2024.5 [30]. Primer sequences (ITS1-30: GTCCCTGCCCTTTGTACACA; ITS1-217R: TTTCGCTGCGTTCTTCATCG) were removed using Cutadapt version 5.2 [44], including trimming of both forward and reverse complement adapter sequences to account for read-through in short amplicons, with reads lacking primer matches discarded. Denoising of trimmed *ITS* reads was performed with DADA2 [31] without fixed truncation lengths (trunc-len = 0) to preserve the variable-length *ITS* region. Taxonomic classification of *ITS* ASVs was performed using the UNITE v10 reference database (release 19 February 2025) [45] at the 99% similarity threshold via consensus VSEARCH [46]. Non-fungal ASVs were subsequently removed by retaining only sequences assigned to the kingdom Fungi.

All statistical analyses and data visualization were performed in R version 4.3.1 [47] using the packages tidyverse v2.0.0 [48], vegan v2.6-4 [49], and ggplot2 v3.4.2 [50]. Prior to alpha- and beta-diversity analyses, the ASV table was rarefied to the minimum library size using the rrarefy function implemented in the *vegan* package in R. For the bacterial (16S) dataset, alpha diversity, beta diversity, taxonomic relative abundance profiles, and genus-level heatmaps were computed and visualized as described below. For the fungal (*ITS*) dataset, analyses were limited to alpha diversity, beta diversity, and taxonomic relative abundance profiles.

Alpha diversity was assessed to characterize microbial community richness and diversity within samples. Four metrics were calculated: observed ASVs, representing the number of unique ASVs detected per sample and calculated using the specnumber function [49]; Chao1 richness estimator, calculated using the estimateR function from the vegan package [51]; Shannon diversity index, calculated using the *diversity* function with the index set to “shannon” [52]; and Good’s coverage, calculated as *C* = 1 − (*n*1/*N*), where *n*1 is the number of singleton ASVs and *N* is the total number of sequences in a sample [53]. Alpha diversity was assessed using four complementary metrics capturing distinct aspects of community structure and sequencing completeness. Observed ASVs provide a direct count of unique amplicon sequence variants detected in each sample but are sensitive to sequencing depth. Chao1 corrects for undetected rare taxa using singleton and doubleton frequencies, offering a more robust richness estimate for species-rich soil communities [51]. Shannon’s diversity index integrates both richness and evenness, reflecting shifts in dominance patterns that richness-only metrics may miss [52]. Good’s coverage served as a sequencing completeness control, confirming that rarefaction depth was sufficient to capture most of the detectable community diversity [53]. The combined use of these metrics reduces dependence on any single index and provides a multi-dimensional characterization of microbial diversity along the salinity gradient [54]. Statistical differences in alpha diversity indices among salinity groups were evaluated using Kruskal–Wallis tests. Alpha diversity boxplots were generated using ggplot2 [50], with individual samples displayed as jittered points.

Beta diversity was assessed to compare microbial community composition among samples using Bray–Curtis dissimilarity matrices, calculated with the vegdist function from the vegan package [49]. Analysis of Similarity (ANOSIM) was performed with 999 permutations using the anosim function [49] to test for significant differences in community structure among salinity groups [55]. Principal Coordinates Analysis (PCoA) was conducted using the cmdscale function to visualize differences in microbial community composition in two-dimensional ordination space [56]. The proportion of variance explained by each principal coordinate axis was calculated from the corresponding eigenvalues. PCoA plots were visualized using ggplot2 [50].

Relative abundance profiles were calculated by converting ASV counts to percentages by dividing each ASV count by the total number of sequences per sample and multiplying by 100. Taxonomic composition at the class and order levels was visualized using bar plots generated with ggplot2 [50]. To identify chemical parameters significantly shaping bacterial community composition, environmental variables were fitted onto the PCoA ordination using the envfit function implemented in the vegan package v2.6-4 in R [49,56]. The significance of each environmental vector was assessed using permutation tests (999 permutations), and the coefficient of determination (r^2^) was used to quantify the strength of the association between individual chemical parameters and microbial community structure. Only parameters showing statistically significant associations (*p* < 0.05) were considered in the interpretation of ordination results. Heatmaps at the genus level were constructed using pheatmap v1.0.12 [57] to illustrate patterns in microbial community composition across samples. Abundance values were log_10_(x + 1)-transformed to stabilize variance and reduce the influence of highly abundant taxa [58]. Samples in heatmaps were ordered according to environmental parameters, including chloride concentration, sodium concentration, sulfate concentration, and total mineralization, to facilitate the identification of relationships between microbial taxa and environmental factors.

## 3. Results

### 3.1. Soil Chemical Characteristics Along a Salinity Gradient

The classification of soils according to the degree of salinity was carried out according to Richards (1954) [28] and FAO (2021) [59]. Additionally, the ionic composition of the water extract was taken into account, which made it possible to determine the type of salinity (chloride, sulfate, soda, and mixed). For this, we analyzed the ratio of base cations (Ca^2+^, Mg^2+^, Na^+^, K^+^) and anions (Cl^−^, SO_4_^2−^, HCO_3_^−^, CO_3_^2−^) in the soil solution. The predominant ions determine the geochemical type of salinity and its genesis, which is widely used in soil-ecological studies of arid regions.

The use of this classification makes it possible to compare the results of various studies and is widely used in the study of soil microbial communities in saline ecosystems, since the level of salinity is one of the key factors determining the structure and functional diversity of the soil microbiome. In our studies, the classification of soil salinity was carried out according to this scale based on indicators of water extraction and dry residue (Table 1).

The chemical properties of the analyzed soils (*n* = 20) reflected a wide range of saline–alkaline conditions as defined by internationally accepted salinity classifications. Soil pH (H_2_O) values were consistently alkaline, ranging from moderately to highly alkaline (7.91–10.47), indicating carbonate–alkaline buffering across all samples (Table 1, Table S2).

Total mineralization, expressed as dense (dry) residue, varied by more than two orders of magnitude, from 256 to 26,312 mg/L. Based on these values and established international thresholds, soils were classified as non-saline (*n* = 3), moderately saline (*n* = 3), and highly saline (*n* = 14). Non-saline soils were characterized by dry residue values below 400 mg/L, moderately saline soils ranged between approximately 400 and 2000 mg/L, while highly saline soils consistently exceeded 2000 mg/L. This salinity gradient represented the primary axis of chemical differentiation among the samples.

Organic matter content was generally low, being classified as very low or low in the majority of soils, and showed limited variability compared to mineral parameters. In contrast, nutrient-related indicators (NO_3_^−^-N, P_2_O_5_, K_2_O) exhibited substantial variability but did not follow the salinity gradient consistently and therefore contributed less to large-scale soil differentiation.

The ionic composition of the water extracts indicated that salinity was predominantly driven by sodium in association with chloride and sulfate anions. Sodium was the dominant soluble cation across most samples, with concentrations increasing markedly from non-saline to highly saline soils. Chloride and sulfate showed the highest variability among anions and largely determined the classification of soils as chloride–sulfate or sulfate–chloride types. Bicarbonate concentrations were elevated in several samples and were associated with high pH values, contributing to mixed anionic compositions but showing lower variability than chloride and sulfate.

Ratios of Na^+^ to divalent cations (Ca^2+^ and Mg^2+^) increased along the salinity gradient, indicating a shift toward sodium-dominated soil solutions in highly saline soils. Potassium concentrations in water extracts were generally low relative to sodium and did not show a consistent relationship with total mineralization. Overall, the combined patterns of total mineralization and ionic composition revealed pronounced chemical heterogeneity among the studied soils, with salinity intensity and dominant soluble ions representing the key factors structuring differences between samples.

In this study, the term “salinity gradient” is used to describe the natural variability of the salinity level between different soils located in different soil and geographical conditions of Kazakhstan. It should be noted that this gradient is not an artificially created experimental gradient, but rather a natural sequence of soils with varying degrees of accumulation of soluble salts.

This approach is widely used in soil and environmental studies to study the influence of environmental factors on the structure of microbial communities, when the sample includes soils with different salinity intensities resulting from differences in hydrogeochemical and climatic conditions [60].

Thus, the studied soils represent a natural ecological salinity gradient reflecting the spatial heterogeneity of salt accumulation processes in arid and semiarid regions.

### 3.2. Alpha Diversity of Soil Microbial Communities Along the Salinity Gradient

**Figure 2:**
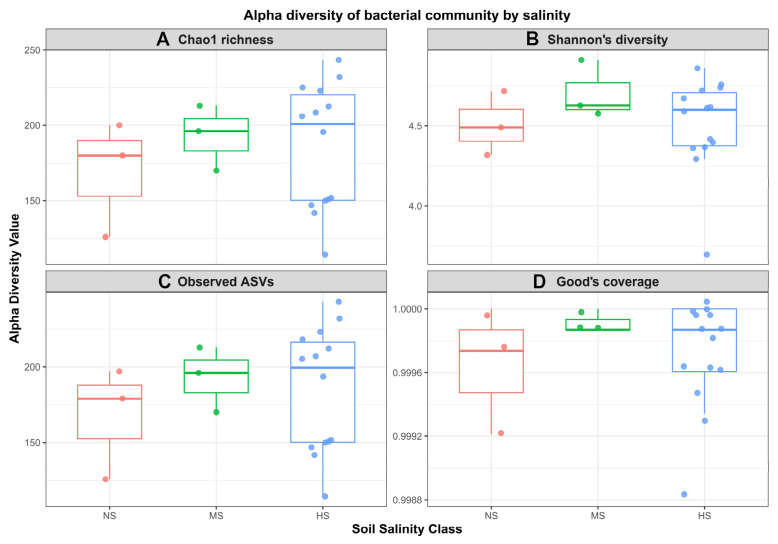
Alpha diversity of soil microbial communities across salinity classes. (**A**) Chao1 richness, (**B**) Shannon’s diversity, (**C**) observed ASVs, and (**D**) Good’s coverage in non-saline (NS), moderately saline (MS), and highly saline (HS) soils.

The alpha diversity of soil bacterial communities across non-saline (NS), moderately saline (MS), and highly saline (HS) soils revealed no significant differences among salinity classes (Figure 2). Richness-related indices (Chao1 and observed ASVs) showed comparable median values among salinity categories, with substantial overlap of interquartile ranges across all groups. Notably, within-group variability was most pronounced in the HS soils, as reflected by a wider dispersion of values for both Chao1 and observed ASVs (Figure 2A,C).

Shannon’s diversity index, which integrates both richness and evenness components, exhibited minimal variation in median values among NS, MS, and HS soils. The extensive overlap of interquartile ranges indicates the absence of clear differentiation in community diversity based on this index across salinity classes (Figure 2B).

Sequencing depth was sufficient across all samples, as indicated by consistently high Good’s coverage values (>0.99) with minimal variability among salinity groups, confirming that the observed alpha diversity patterns were supported by sufficient sequencing depth (Figure 2D).

### 3.3. Beta Diversity of Soil Microbial Communities

**Figure 3:**
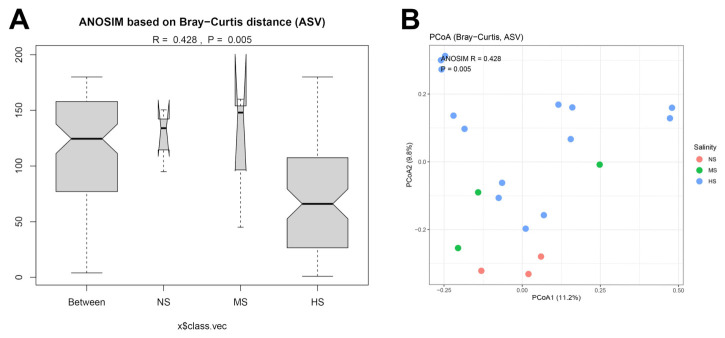
Beta diversity of soil bacterial communities across salinity classes. (**A**) Analysis of similarities (ANOSIM) based on Bray–Curtis dissimilarities calculated from ASV-level relative abundance data, showing a significant difference in community composition among non-saline (NS), moderately saline (MS), and highly saline (HS) soils (R = 0.428, *p* = 0.005). (**B**) Principal Coordinates Analysis (PCoA) ordination based on Bray–Curtis distances. The first two axes explain 11.2% (PCoA1) and 9.8% (PCoA2) of the total variation in bacterial community composition. Points are colored according to salinity class.

Statistically significant differences in soil microbial community composition were detected among non-saline (NS), moderately saline (MS), and highly saline (HS) soils based on ANOSIM using Bray–Curtis dissimilarities at the ASV level (R = 0.428, *p* = 0.005; Figure 3A). The magnitude of the R statistic indicates moderate differentiation among salinity classes while also reflecting substantial within-group variability.

These patterns were further illustrated by Principal Coordinates Analysis (PCoA; Figure 3B). The first two axes explained 11.2% (PCoA1) and 9.8% (PCoA2) of the total variance in community composition. The ordination revealed partial separation of samples according to salinity class; however, considerable overlap among NS, MS, and HS groups was observed, and no distinct or discrete clustering of microbial communities was evident, consistent with the moderate level of differentiation indicated by ANOSIM.

**Figure 4:**
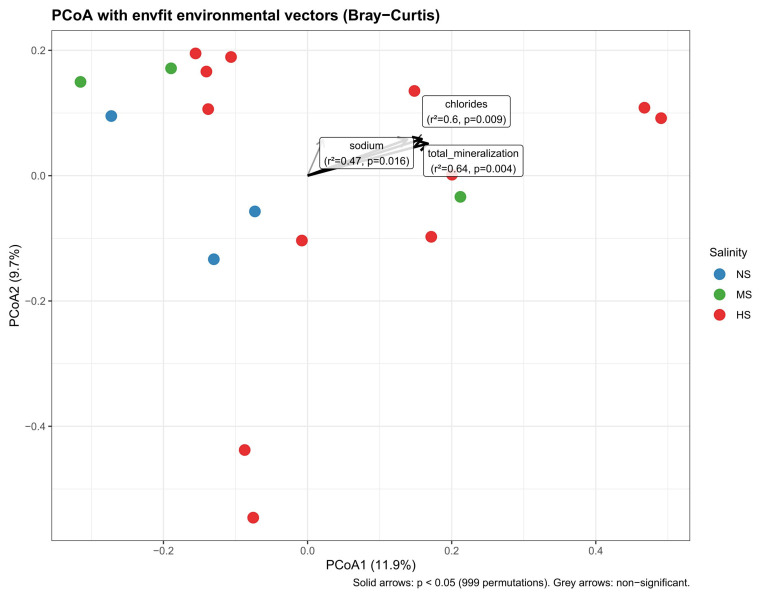
Principal coordinates analysis (PCoA) of bacterial community composition based on Bray–Curtis dissimilarity. Vectors indicate chemical parameters significantly associated with community structure, fitted using the envfit procedure (999 permutations; *p* < 0.05). Vector length is proportional to the strength of the association (r^2^). NS, non-saline soils; MS, moderately saline soils; HS, highly saline soils.

Envfit analysis identified total mineralization (r^2^ = 0.64, *p* = 0.004), chloride (r^2^ = 0.60, *p* = 0.009), and sodium (r^2^ = 0.47, *p* = 0.016) as significant predictors of bacterial community composition (Figure 4). In contrast, sulphate concentration was not a significant predictor at the whole-community level (r^2^ = 0.20, *p* = 0.558), despite its association with specific taxa such as Thermodesulfobacteriota. Together, these results indicate that overall salt load, driven primarily by Na^+^ and Cl^−^, represents the main chemical axis structuring bacterial communities across the salinity gradient.

### 3.4. Comparison of Phylum-Level Microbial Communities Among Soil Salinity Classes

A total of 39 prokaryotic phyla were identified across the 20 analyzed soil samples. The microbial communities exhibited a pronounced dominance structure. The three most abundant phyla—Actinomycetota, *Bacteroidota*, and Pseudomonadota —showed mean relative abundances of 26.63%, 21.47%, and 19.71%, respectively, with individual sample values ranging from 5.34% to 51.77%. Collectively, these three phyla accounted for 48.7% to 85.8% of the total relative abundance of the microbial community in individual samples (Table 1 and Tables S3 and S4; Figure 3). A group of subdominant but consistently detected taxa included “Candidatus Patescibacteria”, Bacillota (formerly Firmicutes), Acidobacteriota, Gemmatimonadota, Chloroflexota, Verrucomicrobiota, Planctomycetota, and Nitrospirota, whose mean relative abundances ranged from 5.53% to 1.06%. Taken together, the 11 most abundant phyla (dominant and subdominant) contributed 89.6% to 100% of the total relative abundance in individual samples, indicating their predominant role in shaping the overall microbial community structure. The cumulative contribution of the remaining 28 phyla was relatively low in most samples; however, individual taxa in certain soils locally reached several percent of relative abundance. Overall, these phyla were characterized by irregular distribution and low detection frequency and are fully presented in Supplementary Table S3.

Comparative analysis conducted for the 11 dominant and subdominant bacterial phyla revealed differences in their relative abundances among soils classified into distinct salinity classes (NS, MS, and HS). Mean relative abundance values, together with the results of statistical testing using the Kruskal–Wallis test with Benjamini–Hochberg correction, are summarized in Table 2. Bacteroidota exhibited higher mean relative abundance in saline soils (HS) compared with non-saline (NS) and moderately saline (MS) soils. In contrast, Acidobacteriota showed higher mean relative abundances in non-saline and moderately saline soils, with reduced contributions in saline soils. Chloroflexota reached their highest mean relative abundance in moderately saline soils, whereas lower values were observed in both non-saline and saline soils. In contrast, Actinomycetota and Pseudomonadota maintained comparable relative abundance levels across all salinity classes and remained dominant components of the microbial communities regardless of soil type. The remaining subdominant phyla displayed substantial inter-sample variability, which was reflected in differences in their mean relative abundances among salinity classes.

Among bacterial phyla with low overall relative abundance (mean < 1%), several significant associations with individual chemical parameters were detected. In particular, Thermodesulfobacteriota showed a significant positive correlation with sulfate concentration (SO_4_^2−^, mg L^−1^; Spearman’s ρ = 0.66, *p* = 0.0022, FDR q = 0.042), indicating a consistent increase in relative abundance with increasing sulfate levels across samples. In addition, Cyanobacteriota, another low-abundance phylum, exhibited a significant negative association with chloride concentration (Cl^−^; ρ = −0.68, *p* = 0.0013, FDR q = 0.039). No other low-abundance phyla demonstrated statistically significant correlations with the examined chemical parameters after correction for multiple testing.

A total of 97 prokaryotic classes were detected across the 20 soil samples based on the full class-level dataset. For the main summary (Table 3), we report 27 classes with Total mean ≥0.5%, which together accounted for 84.67–97.96% of the total relative abundance at the class level across individual samples. Community composition was dominated by Bacteroidia (mean 17.22%, range 3.62–39.55%), followed by Acidimicrobiia (16.72%, 0.68–39.62%) and Gammaproteobacteria (12.93%, 1.37–46.99%), with additional contributions from Actinobacteria (7.46%, 0.76–15.42%) and Alphaproteobacteria (6.79%, 0.14–24.27%) (Table 3 and Tables S5 and S6).

Comparisons among salinity classes (NS, MS, HS) did not identify any class with statistically significant differences after Benjamini–Hochberg correction (FDR q < 0.05; Table 3). The smallest nominal *p*-values prior to correction were observed for Vicinamibacteria (*p* = 0.0039; q = 0.097), Bacteroidia (*p* = 0.0072; q = 0.097), and Rhodothermia (*p* = 0.0113; q = 0.102), but these did not remain significant following FDR adjustment. In contrast, correlation analyses with continuous chemical parameters revealed several statistically significant class-level associations.

Rhodothermia showed strong positive correlations with total dissolved solids (TDS), sodium (Na^+^), and chloride (Cl^−^), whereas Vicinamibacteria exhibited consistent negative associations with the same parameters. Among additional classes included in Table 3, Cyanobacteriia and Holophagae displayed significant negative correlations with chloride concentration, while Desulfuromonadia showed concordant patterns with sulfate-enriched samples, consistent with phylum-level observations. Notably, these chemical associations were detected for a subset of classes despite the absence of statistically significant differences among categorical salinity classes.

### 3.5. Genus-Level Microbial Community Patterns Along Salinity-Associated Chemical Gradients

**Figure 5:**
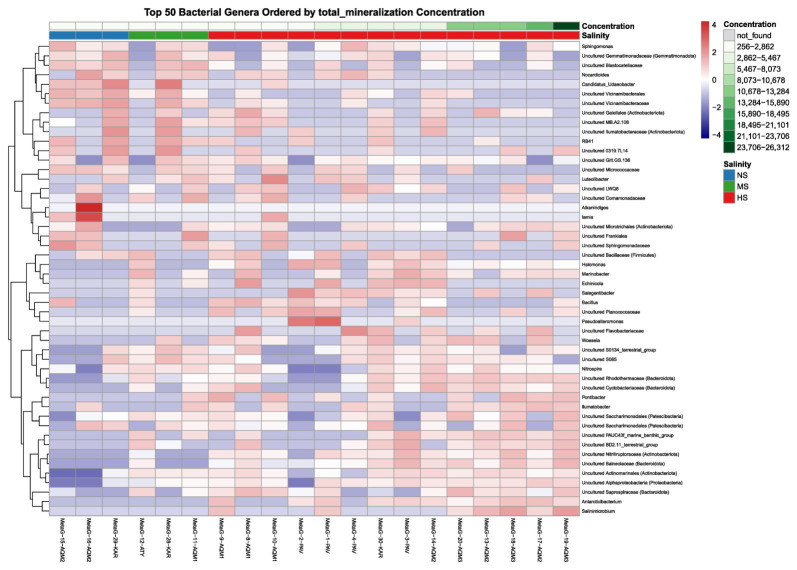
Genus-level heatmap of soil bacterial communities ordered along the total mineralization gradient. Heatmap showing standardized relative abundance (z-scores) of the top 50 bacterial genera across samples ordered by increasing total mineralization. This integrative gradient captures the combined effects of salinity-related chemical factors and reveals the most pronounced and coherent genus-level community shifts.

**Figure 6:**
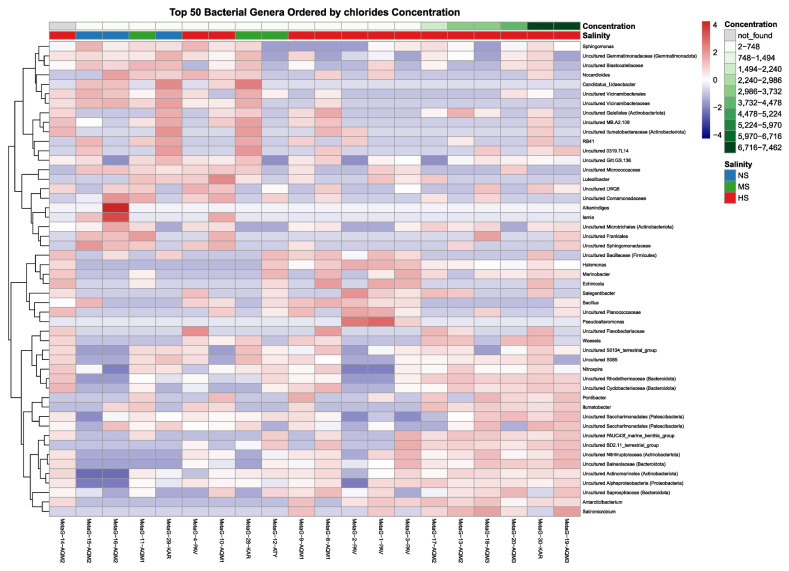
Genus-level heatmap of soil bacterial communities ordered along the chloride (Cl^−^) gradient. Heatmap showing the relative abundance (z-score normalized) of the top 50 most prevalent bacterial genera across soil samples ordered by increasing chloride (Cl^−^) concentration (from low to high). Columns represent individual samples; rows correspond to bacterial genera. Color intensity reflects standardized relative abundance. Salinity classes (NS, MS, HS) and chloride concentration ranges are indicated above the heatmap.

**Figure 7:**
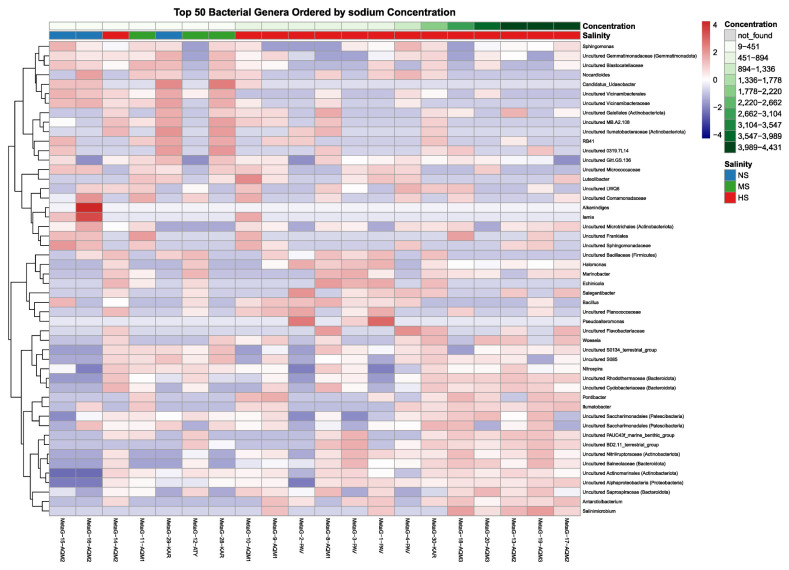
Genus-level heatmap of soil bacterial communities ordered along the sodium (Na^+^) gradient. Heatmap of the top 50 most prevalent bacterial genera across samples ordered by increasing sodium (Na^+^) concentration. Relative abundances are shown as z-score-standardized values. Salinity class annotations (NS, MS, HS) are overlaid to illustrate their distribution along the continuous sodium gradient.

**Figure 8:**
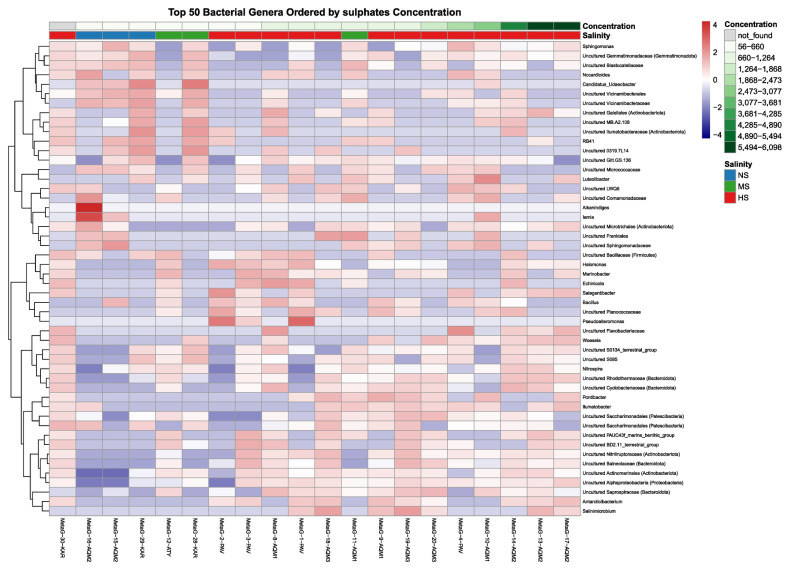
Genus-level heatmap of soil bacterial communities ordered along the sulphate (SO_4_^2−^) gradient. Heatmap illustrating genus-level relative abundance patterns (z-score normalized) of the 50 most prevalent bacterial taxa across samples ordered by increasing sulphate (SO_4_^2−^) concentration. The absence of consistent directional shifts highlights taxon-specific and heterogeneous responses to sulphate variation.

Genus-level heatmaps constructed for the 50 most prevalent bacterial taxa revealed pronounced, gradient-dependent shifts in community composition when samples were ordered along continuous gradients of chloride (Cl^−^), sodium (Na^+^), sulphate (SO_4_^2−^), and total mineralization (Figure 5, Figure 6, Figure 7 and Figure 8). Across all gradients, the bacterial communities were phylogenetically diverse and were dominated by members of Pseudomonadota (particularly the classes Alphaproteobacteria and Gammaproteobacteria), Actinomycetota, Acidobacteriota, Bacteroidota, Gemmatimonadota, Chloroflexota, and Verrucomicrobiota.

Several genera, including *Sphingomonas*, uncultured representatives of Gemmatimonadaceae, Nanosynbacterales, Chloroflexota (g__Gitt.GS.136), Gemmatimonadota (g__S0134_terrestrial_group), and Dehalococcoidia (g__S085), exhibited stable occurrence and heterogeneous relative abundance across samples spanning a wide range of chloride (Cl^−^), sodium (Na^+^), sulphate (SO_4_^2−^), and total mineralization levels. The absence of consistent monotonic trends along these chemical gradients suggests that these taxa constitute a broadly distributed core assemblage with limited sensitivity to salinity-related chemical variation.

When samples were ordered along gradients of chloride (Cl^−^), sodium (Na^+^), and total mineralization, the genera *Salinimicrobium* and *Antarcticibacterium*, representatives of Nitriliruptoraceae, uncultured genera within the families Saprospiraceae and Balneolaceae, as well as two members of Gemmatimonadota (g__PAUC43f_marine_benthic_group and g__BD2.11_terrestrial_group), showed increasing relative abundance toward higher concentrations, as reflected by persistently positive z-score values. The clearest and most coherent pattern was observed when samples were ordered along the total mineralization gradient.

In contrast, an inverse relationship with chloride, sodium, and total mineralization gradients was observed for two genera affiliated with the order Vicinamibacterales, which exhibited higher relative abundance under low-salinity conditions. In addition, increased relative abundance of “Candidatus Udaeobacter” (family Chthoniobacteraceae) was associated with low levels of total mineralization.

Unlike chloride and sodium, ordering the 50 most prevalent bacterial taxa along the sulphate gradient revealed a more heterogeneous and taxon-specific response pattern, without the emergence of clear or consistent associations with sulphate concentration.

### 3.6. Fungal Community Diversity Along the Salinity Gradient

Prior to downstream analyses, four samples (ITS-3, ITS-8, ITS-12, and ITS-16) did not yield detectable fungal ITS sequences and were therefore excluded from fungal community analyses. Three of these samples (ITS-3, ITS-8, ITS-12) belonged to the highly saline (HS) group, whereas one sample (ITS-16) was classified as non-saline (NS).

The absence of detectable fungal signal in these samples may reflect a combination of low fungal biomass and technical limitations associated with ITS amplification in soils with extreme physicochemical properties. In particular, highly saline conditions are known to reduce fungal abundance and DNA recoverability. In the case of the non-saline sample (ITS-16), the lack of fungal detection may be related to its low humus content, potentially indicating reduced microbial biomass or uneven spatial distribution of fungi within the soil matrix.

#### 3.6.1. Alpha Diversity of Fungal Communities

**Figure 9:**
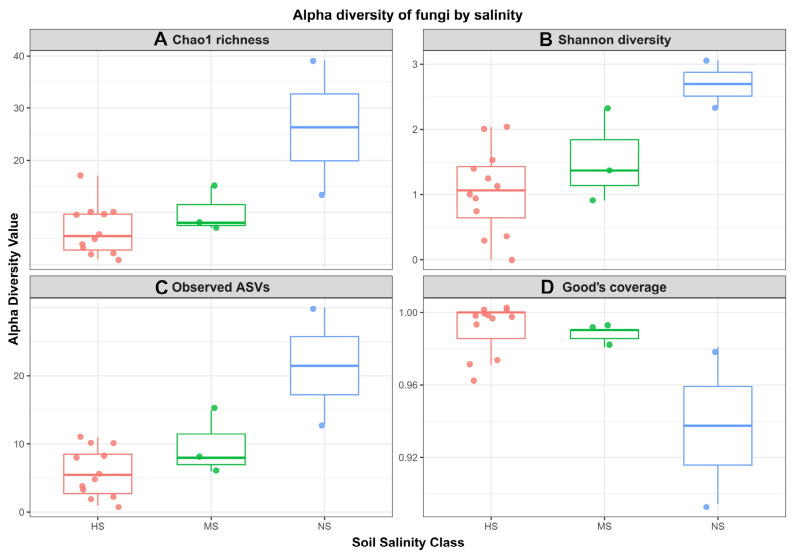
Alpha diversity of soil fungal communities across salinity classes. (**A**) Chao1 richness, (**B**) Shannon diversity, (**C**) observed ASVs, and (**D**) Good’s coverage in non-saline (NS), moderately saline (MS), and highly saline (HS) soils.

Alpha diversity of soil fungal communities, assessed using Chao1 richness, observed ASVs, Shannon’s diversity index, and Good’s coverage, showed no statistically significant differences among non-saline (NS), moderately saline (MS), and highly saline (HS) soils (Figure 9).

Richness-based indices (Chao1 and observed ASVs) exhibited comparable median values across all salinity classes, with substantial overlap of interquartile ranges. Although slightly higher median richness values were observed in non-saline soils, this trend was not consistent and remained within the range of intra-group variability.

Shannon’s diversity index showed moderate variation among samples but did not display any directional trend along the salinity gradient. Good’s coverage values were consistently high (>0.97), indicating sufficient sequencing depth across all samples.

Overall, these results indicate that fungal alpha diversity remains stable across the examined salinity gradient.

#### 3.6.2. Beta Diversity of Fungal Communities

**Figure 10:**
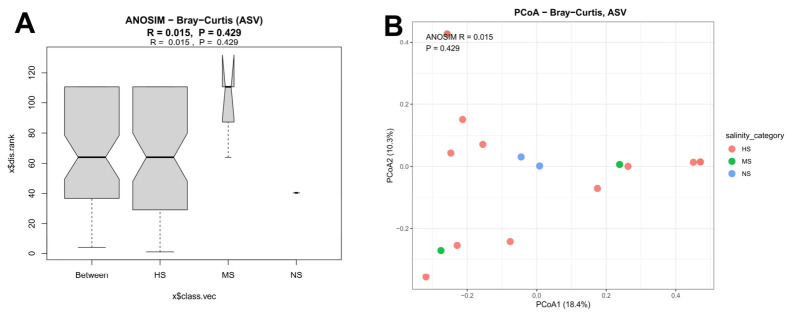
Beta diversity of soil fungal communities. (**A**) ANOSIM based on Bray–Curtis dissimilarities (R = 0.015, *p* = 0.429). (**B**) PCoA ordination of fungal communities. Points are colored by salinity class.

Analysis of similarities (ANOSIM) based on Bray–Curtis dissimilarities at the ASV level revealed no statistically significant differentiation in fungal community composition among salinity classes (R = 0.015, *p* = 0.429; Figure 10A).

The near-zero R value indicates that variation within salinity classes is comparable to variation between classes, suggesting the absence of salinity-driven structuring of fungal communities.

This pattern was further supported by Principal Coordinates Analysis (PCoA), where samples from NS, MS, and HS soils showed extensive overlap without distinct clustering (Figure 10B). The first two axes explained 18.4% (PCoA1) and 10.3% (PCoA2) of total variance, indicating that the major drivers of fungal community variation are not associated with salinity.

Together, these results demonstrate that, in contrast to bacterial communities, fungal community composition is not significantly structured by salinity under the conditions studied.

#### 3.6.3. Phylum-Level Composition of Fungal Communities

**Figure 11:**
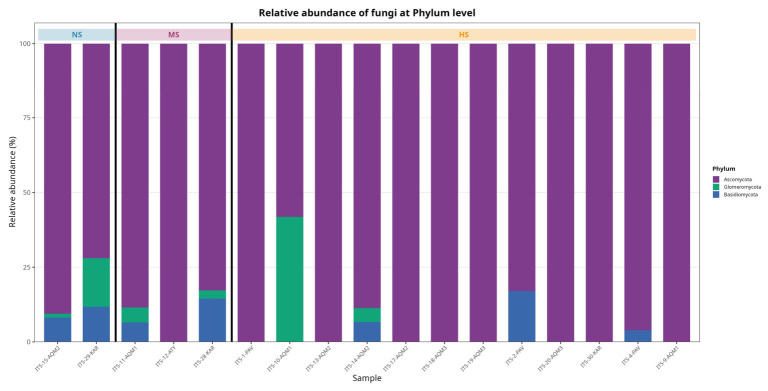
Relative abundance of fungal communities at the phylum level across samples grouped by salinity class.

Fungal communities across all samples were strongly dominated by Ascomycota, which accounted for the majority of sequences irrespective of salinity class (Figure 11). In most samples, the relative abundance of Ascomycota exceeded 80–95%, indicating a highly consistent dominance pattern across the entire gradient.

Minor contributions from Basidiomycota and Glomeromycota were observed in a limited subset of samples, without any consistent association with salinity class. The presence of these phyla was sporadic and characterized by substantial inter-sample variability.

The pronounced and stable dominance of Ascomycota suggests that fungal community structure at the phylum level is largely conserved across the examined salinity gradient and is not strongly influenced by increasing salt concentration.

#### 3.6.4. Genus-Level Composition of Fungal Communities

**Figure 12:**
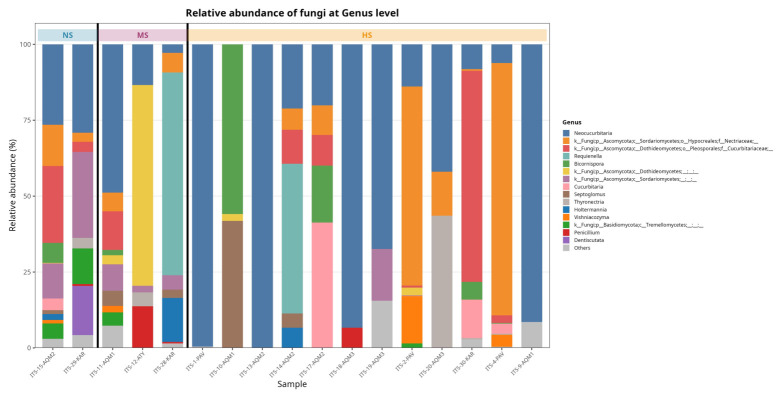
Relative abundance of fungal communities at the genus level across samples grouped by salinity class.

At the genus level, fungal communities showed pronounced inter-sample variability, with several samples dominated by one or a few genus-level taxa (Figure 12). The most abundant taxon across the dataset was *Neocucurbitaria*, which was detected in almost all samples and occurred across non-saline, moderately saline, and highly saline soils. In several highly saline samples, *Neocucurbitaria* represented the dominant component of the fungal community.

Other relatively abundant genus-level assignments included unclassified members of *Nectriaceae*, *Cucurbitariaceae*, *Dothideomycetes*, and *Sordariomycetes*, as well as *Requienella*, *Bicornispora*, *Septoglomus*, *Thyronectria*, *Holtermannia*, *Vishniacozyma*, *Penicillium*, and *Dentiscutata*. However, these taxa were unevenly distributed and often showed high abundance only in individual samples.

Overall, fungal genus-level composition did not show a clear salinity-class-specific pattern. Dominant genera and genus-level groups occurred across different salinity classes, and the observed variation was mainly driven by individual sample differences rather than consistent shifts from non-saline to highly saline soils. These results support the phylum-level pattern, indicating that fungal community composition was relatively weakly structured by the salinity gradient.

## 4. Discussion

In the context of the predicted intensification of soil salinization processes due to climatic changes and anthropogenic impact, the importance of biological approaches to soil fertility management is increasing. Manipulation of the composition and functional structure of soil microbial communities is considered as a promising direction for increasing plant resistance to salt stress and ensuring long-term productivity of saline agroecosystems, as well as for bioremediation and restoration of degraded lands [61,62,63,64]. Native (autochthonous) microbial strains isolated from local soils or the rhizosphere of plants often turn out to be more effective inoculates than “universal” collection strains, since they are already adapted to soils and climatic conditions [65,66]. The selection of native microorganisms for the subsequent development of microbiological inoculates should be based on a detailed analysis of the structure of soil and rhizosphere microbiomes. The identification of taxonomic units that are consistently present and enriched in conditions of high salinity makes it possible to purposefully select potentially promising taxa adapted to the aggressive physico-chemical conditions of saline soils [67]. In the framework of this study, the microbiome of 20 soil samples was analyzed, taken in areas not involved in agricultural use, and characterized by a sharply alkaline reaction, low humus content and a pronounced gradient of total mineralization—from normal to extremely saline conditions. The data obtained showed that in the analyzed natural soils of Kazakhstan, formed under conditions of prolonged and chronic salt accumulation, the intensity of salinity and the ionic composition of the soil solution are the main factors determining the structure of microbial communities, while the content of organic matter and available nutrients plays a secondary role. This is confirmed by the high variability of indicators of total mineralization, sodium, chlorides and sulfates, as well as a pronounced alkaline reaction of the medium (pH up to 10.5), which form strict abiotic restrictions for microorganisms. These chemical gradients were directly reflected in microbial community patterns. Stable α-diversity and moderate β-diversity, with increased PCoA dispersion, indicate salinity restructures microbial communities rather than depleting diversity.

The data obtained show that the α-diversity of soil microbial communities remained relatively stable (Chao1, Shannon, and observed ASVs) along the salinity gradient from unsalted to extremely saline soils. This result contrasts with a number of studies performed in agricultural soils, where an increase in salinity was often accompanied by a decrease in both α- and β-diversity of microbial communities [68,69]. In contrast to these studies, the soils we studied were not subjected to agrochemical effects and represented long-lasting natural systems. Probably, under conditions of chronic salt stress, such ecosystems are characterized by a stable restructuring of the structure of microbial communities without a marked loss of taxonomic richness, which may explain the absence of a significant decrease in α-diversity in our study. Similar results were obtained for natural brackish-alkaline groundwater, where the α-diversity indices (Chao1, Shannon, and observed ASVs) remained independent of the salinity gradient, which the authors attribute to the long geological and evolutionary time of adaptation of microbial communities to brackish conditions [70].

At the same time, the preservation of taxonomic diversity does not exclude functional shifts in microbial communities. For saline–saline soils, it has been shown that salt stress can be accompanied by increased enzymatic activity and an increase in the functional diversity of microbial communities, estimated on the basis of profiles of soil enzymes [71]. The moderate but statistically significant differentiation of microbial communities by β-diversity observed in this study indicates that salinity has a directed effect on the composition of soil microbiomes, but does not lead to the formation of clearly delineated types of communities. The partial overlap of the samples in the PCoA space and the moderate values of the difference statistics reflect the gradient nature of the changes rather than the abrupt restructuring of microbial assemblages. These results are consistent with research data covering a wide range of salinity, where β-diversity varies along a salt stress gradient [60,69], whereas the inconsistent effects described earlier are attributed to a limited range of salinity and a small number of observations [72,73,74]. The results of environmental vector fitting further support this interpretation. The envfit analysis demonstrated that total mineralization, chloride, and sodium concentrations were the strongest predictors of whole-community compositional variation, whereas sulphate did not show a significant association at the community-wide level. This indicates that the overall salt load, driven primarily by Na^+^ and Cl^−^, represents the dominant chemical axis structuring bacterial communities across the studied soils. At the same time, the lack of a significant sulphate effect at the whole-community level does not preclude its importance for specific functional groups, such as sulphate-reducing Thermodesulfobacteriota, highlighting a scale-dependent response of microbial taxa to individual ions.

Taken together, these results indicate that the core of the manuscript is not merely the description of microbial taxonomic composition, but the evaluation of how long-term natural salinity and ionic composition shape bacterial and fungal communities in saline–alkaline soils of Kazakhstan. The strongest ecological signal was observed for bacterial communities, whose compositional turnover was associated mainly with total mineralization and Na^+^–Cl^−^-driven salinity, whereas fungal communities showed comparatively weak salinity-related differentiation. Thus, the study links microbial community structure to measured soil physicochemical properties rather than treating community composition as an isolated descriptive pattern.

Consistent with this gradient-driven pattern, salinity does not lead to a complete restructuring of microbial communities, but primarily affects the relative representation of taxa within an already established taxonomic structure. In such conditions, the dominant phylum is expected to remain while communities differentiate at lower taxonomic levels. The dominance of Actinomycetota, Bacteroidota, and Pseudomonadota in all the studied soils, regardless of the salinity level, indicates the presence of a stable taxonomic framework of microbial communities characteristic of arid and alkaline soils. The preservation of these phyla along a wide salinity gradient is consistent with their well-known ecological plasticity, metabolic diversity, and ability to adapt to osmotic and ionic stress conditions [75].

The ion-associated patterns observed in this study are consistent with well-established differences in osmoadaptive strategies among dominant soil phyla. Most representatives of Actinomycetota and Pseudomonadota rely on a “low-salt-in” strategy, maintaining low intracellular NaCl concentrations through active ion homeostasis while accumulating organic compatible solutes such as glycine betaine, ectoine and hydroxyectoine [76,77]. This form of osmoadaptation allows dynamic adjustment to fluctuating salinity while preserving protein stability and cellular function without extensive proteome reorganization. In contrast, a subset of *Bacteroidota*, exemplified by highly halophilic taxa such as *Salinibacter ruber*, employs a “high-salt-in” strategy characterized by the accumulation of molar KCl concentrations and a proteome intrinsically adapted to high ionic strength. This ion-based mode of adaptation is associated with a highly acidic protein composition and functionally aligns these bacteria with extremely halophilic archaea [77,78].

Despite the presence of *Bacteroidota* in all types of soils studied, their higher relative abundance was observed in highly saline samples compared to unsalted and moderately saline soils, while representatives of *Acidobacteriota* demonstrated higher average relative abundance in unsalted and moderately saline conditions. The enrichment of *Bacteroidota* in saline soils revealed in this study is consistent with the results obtained earlier for naturally saline ecosystems [79,80,81]. This pattern is supported by published data showing that many halotolerant *Bacteroidota* possess efficient salt-out osmoadaptation strategies, including accumulation of compatible solutes and extracellular polymeric substances, which mitigate Na^+^ and Cl^−^ toxicity and facilitate persistence under high ionic strength, whereas *Acidobacteriota* generally lack such traits and preferentially occur under low-salinity conditions [82]. The decrease in the relative abundance of *Acidobacteriota* with an increase in salinity observed in this study is consistent with the data obtained earlier for various soil systems, where representatives of this phylum showed a preference for unsalted or moderately saline conditions [68,83], which makes it possible to consider Acidobacteriota as a sensitive biological indicator of changes in the salt regime of soils [84].

In addition to the taxa showing a clear positive or negative relationship with salinity gradients, a group of genera was identified in the studied soils, characterized by a stable presence and high variability in relative abundance over the entire range of concentrations of Cl^−^, Na^+^, SO_4_^2−^ and total mineralization. Such taxa include representatives of the genera *Sphingomonas*, *Saccharimonadales*, uncultivated representatives of Gemmatimonadaceae, Chloroflexota (g__Gitt.GS.136), Gemmatimonadota (g__S0134_terrestrial_group) and Dehalococcoidia (g__S085). The absence of pronounced monotonous trends for these taxa indicates the presence of a widespread *core assemblage* characterized by high ecological plasticity and relatively low sensitivity to changes in the salt regime [85,86].

At the same time, a directional association with increased concentrations of Cl^−^, Na^+^ and total mineralization was revealed for a number of genera, which was most clearly manifested when the samples were ordered along the gradient of total mineralization. In particular, an increase in the relative abundance of *Salinimicrobium*, *Antarcticibacterium*, representatives of *Nitriliruptoraceae*, as well as uncultivated genera of the *Saprospiraceae* and *Balneolaceae* families, indicates their preference for conditions of increased ion load. Such taxa are traditionally considered as halotolerant or halophilic components of microbial communities adapted to osmotic stress and high concentrations of sodium and chlorides. Experimental and genomic studies indicate that these genera typically combine compatible-solute-based osmoprotection (e.g., trehalose, ectoine or glycine betaine) with active ion transport systems, explaining their positive associations with Na^+^, Cl^−^ and total mineralization observed along continuous salinity gradients [80,87,88,89,90].

On the contrary, representatives of the order *Vicinamibacterales* and the genus “Candidatus Udaeobacter” demonstrated increased relative abundance in conditions of low mineralization, which is consistent with their well-known ecological strategy focused on oligotrophic and less stressful soil conditions [91].

It is noteworthy that, unlike the chloride and sodium gradients, sulfate did not form clear and consistent taxonomic patterns at the level of genera. This indicates a more complex and context-dependent nature of the SO_2_ effect, probably related to local conditions of the redox regime, availability of organic substrate and microbial metabolic interactions, rather than a direct osmotic effect. In particular, representatives of the phylum Thermodesulfobacteriota demonstrated a significant positive correlation with the concentration of sulfates, which is consistent with their functional role in sulfate reduction processes and emphasizes the selective effect of SO_4_^2−^ on individual functional groups of microorganisms [92].

Collectively, these results indicate that soil salinity modulates microbial communities primarily through gradient-driven shifts in the relative abundance of individual taxa. This process promotes salt-tolerant genera while preserving a stable core microbiome, consistent with the moderate β-diversity differentiation observed.

In contrast to bacterial communities, fungal communities did not exhibit significant variation in either alpha or beta diversity across the salinity gradient. The absence of compositional differentiation (ANOSIM R = 0.015, *p* = 0.429) suggests that salinity is not a primary driver of fungal community structure in the studied soils.

This divergence between bacterial and fungal responses highlights fundamental differences in the ecological responses of prokaryotes and unicellular eukaryotes to salinity stress. While bacterial communities are typically structured by deterministic environmental filtering driven by salinity and associated ionic gradients, fungal communities are more strongly influenced by spatial processes and stochastic assembly mechanisms, resulting in greater compositional stability under salinity stress [93]. Consistent with this pattern, fungi have been shown to exhibit higher tolerance to osmotic stress through physiological adaptations, particularly osmoregulatory mechanisms mediated by conserved signaling pathways such as the high-osmolarity glycerol (HOG) pathway [94,95]. These mechanisms enable fungi to maintain cellular homeostasis under hyperosmotic conditions by regulating intracellular solute concentrations and water balance. In addition, filamentous growth and hyphal nutrient foraging may further contribute to the buffering against environmental stress by allowing fungi to exploit spatially heterogeneous resources and maintain metabolic activity under unfavorable conditions [96].

The consistent dominance of Ascomycota across all samples aligns with global observations demonstrating that a limited number of Ascomycota taxa dominate soil fungal communities across diverse ecosystems, including arid and saline environments [97]. The lack of detectable shifts in fungal community structure may reflect physiological and ecological adaptations, including osmoregulation, accumulation of compatible solutes, and the structural buffering capacity of mycelial networks, which enhance tolerance to osmotic stress [95,98].

Furthermore, the absence of clustering in ordination space suggests that local-scale heterogeneity and other environmental factors may exert a stronger influence on fungal communities than bulk salinity. In dryland and stress-prone ecosystems, fungal community assembly is often governed by spatial heterogeneity and resource availability rather than single abiotic gradients [99].

Another limitation of this study is that the amplicon-based *16S rRNA* gene and *ITS* approach provides information on taxonomic composition and diversity, but does not directly measure functional gene abundance or gene expression. Therefore, functional genes involved in carbon and nitrogen cycling were not assessed in the present study. Future work using shotgun metagenomics, metatranscriptomics, or targeted qPCR assays will be required to determine whether the observed taxonomic shifts correspond to changes in functional potential or active biogeochemical processes.

A potential limitation is that DNA extraction relied on enzymatic lysis without mechanical disruption, which may lead to underrepresentation of certain taxa with highly resistant cell walls. However, because all samples were processed using the same standardized protocol, the resulting data remain fully comparable across sites, ensuring the robustness of comparative analyses.

## 5. Conclusions

This study provides a comprehensive characterization of bacterial community responses to long-term natural salinity gradients in saline–alkaline soils of Kazakhstan. Across a broad range of total mineralization (256–26,312 mg/L) and consistently alkaline conditions (pH up to 10.47), bacterial alpha diversity (Chao1, Shannon, observed ASVs) remained relatively stable, indicating that chronic salinity does not necessarily reduce taxonomic richness in natural, non-agricultural soils.

In contrast, beta diversity analyses revealed moderate but statistically significant compositional differentiation among salinity classes (ANOSIM R = 0.428, *p* = 0.005), reflecting gradual community turnover along the salinity gradient rather than abrupt restructuring. Salinity intensity and ionic composition—particularly sodium, chloride, and sulfate—represented the primary environmental drivers shaping microbial community structure.

Highly saline soils were characterized by increased relative abundance of *Bacteroidota*, while *Acidobacteriota* were more prevalent in non-saline and moderately saline conditions, suggesting differential sensitivity and adaptation strategies among major bacterial lineages. Correlation analyses further demonstrated ion-specific associations, including positive relationships between Thermodesulfobacteriota and sulfate and negative associations between Cyanobacteriota and chloride concentration.

In contrast to bacterial communities, fungal communities showed no significant response to the salinity gradient, with both alpha and beta diversity remaining stable across all salinity classes. This indicates that, under the studied conditions, fungal community structure is less sensitive to salinity-driven environmental filtering and is likely governed by other ecological factors such as spatial heterogeneity or resource availability. The consistent dominance of Ascomycota further supports the high tolerance and ecological stability of fungal communities in saline–alkaline soils.

Overall, the results indicate that long-term naturally formed saline–alkaline soils of Kazakhstan harbor a stable yet compositionally responsive bacterial microbiome structured primarily by sodium-dominated salinity gradients. These findings contribute to a better understanding of microbial adaptation to chronic ionic stress in arid ecosystems and provide a taxonomic framework for future studies exploring functional potential and biotechnological applications of halotolerant soil microorganisms.

## Figures and Tables

**Table 1:** The physical and chemical indexes of soil.

Samples	Humus, %	pH	Calcium in Water Extract, mg/L	Magnesium in Water Extract, mg/L	Chlorides, mg/L	Sulfates, mg/L	Total Mineralization/Solid/Dry Residue, mg/L	Sodium in Water Extract, mg/L	Bicarbonates, mg/L
MetaG-1-PAV	1.09	10.06	17.17	3.035	589.2	742.8	3365	873	48.8
MetaG-2-PAV	1.17	10.17	13.07	4.053	585	355.3	2325	653.7	73.2
MetaG-3-PAV	1.29	10.47	38.57	5.143	704.4	362.5	4990	797.8	170.8
MetaG-4-PAV	2.92	7.91	140.2	11.82	141.9	2282	3940	1054	18.3
MetaG-8-AQM1	0.69	9.7	16.32	6.856	578.5	722.8	2188	771.5	1200
MetaG-9-AQM1	0.65	8.82	98.29	40.67	467.8	1003	2116	613.5	1000
MetaG-10-AQM1	0.57	8.8	375.6	39.43	147.3	2952	2276	527.4	900
MetaG-11-AQM1	0.55	8.56	338.6	15.24	6.244	966.5	1080	33.78	700
MetaG-12-ATY	0.22	8.84	42.44	33.41	412.5	172.3	928	243.1	600
MetaG-13-AQM2	3.14	8.71	436.4	228.3	3069	5913	11,884	3999	48.8
MetaG-14-AQM2	0.90	8.99	426.9	22.85	not detected	4452	5184	14.29	not detected
MetaG-15-AQM2	0.92	8.77	30.35	2.931	2.488	62.06	256	9.257	not detected
MetaG-16-AQM2	0.18	8.5	22.63	3.512	4.433	55.62	316	10.47	48.8
MetaG-17-AQM2	1.35	8.66	419	66.52	1876	6098	14,184	4431	48.8
MetaG-18-AQM3	1.19	8.95	1024	435.4	3387	761.1	13,104	2898	48.8
MetaG-19-AQM3	1.86	8.15	759.5	1323	7462	1202	26,312	4321	428
MetaG-20-AQM3	2.49	8.37	357.8	623.7	4155	1564	11,252	3839	68
MetaG-28-KAR	2.06	8.9	4.06	3.535	189.9	266.1	932	328.9	48.8
MetaG-29-KAR	2.39	8.64	12.99	4.796	62.72	94.37	448	118.5	428
MetaG-30-KAR	1.03	8.8	65.45	9.624	7097	not detected	4924	1943	68

**Table 2:** Distribution of major bacterial phyla among soil salinity classes.

Phylum	Min–Max (%)	Total Mean (%)	NS Mean (%)	MS Mean (%)	HS Mean (%)	Kruskal–Wallis *p*	FDR q	Occurrence (*n* of 20 Samples)
Actinomycetota	6.02–44.31	26.63	25.56	30.77	25.98	7.296 × 10^−1^	8.408 × 10^−1^	20/20
Bacteroidota	5.34–47.00	21.47	8.15	10.12	26.76	3.287 × 10^−3^	3.616 × 10^−2^	20/20
Pseudomonadota	6.11–51.77	19.71	28.16	16.28	18.64	7.644E × 10^−1^	8.408 × 10^−1^	20/20
“Candidatus Patescibacteria”	0.00–25.31	5.53	1.79	4.87	6.47	3.602 × 10^−1^	6.544 × 10^−1^	19/20
Bacillota	0.00–24.76	5.12	3.35	2.42	6.08	4.759 × 10^−1^	6.544 × 10^−1^	19/20
Acidobacteriota	0.41–17.83	4.98	12.28	9.02	2.55	9.410 × 10^−3^	5.175 × 10^−2^	20/20
Gemmatimonadota	0.06–11.51	4.54	5.43	5.78	4.09	4.569 × 10^−1^	6.544 × 10^−1^	20/20
Chloroflexota	0.08–11.38	3.96	5.53	9.17	2.51	3.232 × 10^−2^	1.185 × 10^−1^	20/20
Verrucomicrobiota	0.00–11.49	2.17	3.05	3.47	1.71	2.210 × 10^−1^	4.862 × 10^−1^	19/20
Planctomycetota	0.07–3.53	1.22	1.1	1.09	1.28	9.422 × 10^−1^	9.422 × 10^−1^	20/20
Nitrospirota	0.00–3.54	1.06	0.61	1.68	1.03	2.138 × 10^−1^	4.862 × 10^−1^	17/20

**Table 3:** Class-level composition of soil microbial communities across salinity classes (Total mean ≥ 0.5%).

Class	Min–Max (%)	Total Mean (%)	NS Mean (%)	MS Mean (%)	HS Mean (%)	Kruskal–Wallis *p*	FDR q	Occurrence (*n* of 20 Samples)
Bacteroidia	3.62–39.55	17.22	7.8	8.01	21.21	7.205 × 10^−3^	9.727 × 10^−2^	20/20
Acidimicrobiia	0.68–39.62	16.72	10.34	18.01	17.81	4.155 × 10^−1^	7.012 × 10^−1^	20/20
Gammaproteobacteria	1.37–46.99	12.93	17.23	10.47	12.53	8.779 × 10^−1^	9.166 × 10^−1^	20/20
Actinobacteria	0.76–15.42	7.46	9.28	8.13	6.93	7.769E × 10^−1^	9.121 × 10^−1^	20/20
Alphaproteobacteria	0.14–24.27	6.79	10.93	5.81	6.11	8.826 × 10^−1^	9.166 × 10^−1^	20/20
Bacilli	0.00–24.76	4.8	3.07	2.32	5.71	5.690 × 10^−1^	7.361 × 10^−1^	19/20
Rhodothermia	0.00–12.78	3.92	0.04	1.15	5.34	1.135 × 10^−2^	1.021 × 10^−1^	18/20
Saccharimonadia	0.00–16.34	3.9	1.72	2.81	4.6	5.691 × 10^−1^	7.361 × 10^−1^	18/20
Blastocatellia	0.00–9.35	2.17	6.01	4.44	0.86	1.021 × 10^−1^	5.697 × 10^−1^	15/20
Verrucomicrobiae	0.00–11.31	2.06	3.05	3.4	1.55	1.182 × 10^−1^	5.697 × 10^−1^	19/20
Gemmatimonadetes	0.00–7.33	1.58	4	2	0.98	2.789 × 10^−1^	6.275 × 10^−1^	17/20
Anaerolineae	0.00–10.38	1.29	0.19	4.32	0.88	3.731 × 10^−1^	7.012 × 10^−1^	17/20
p__Actinomycetota; c__MB-A2-108	0.00–10.42	1.19	2.43	3.58	0.42	4.965 × 10^−1^	7.361 × 10^−1^	9/20
Thermoleophilia	0.00–6.70	1.16	3.23	1.04	0.74	2.321 × 10^−1^	5.697 × 10^−1^	16/20
Vicinamibacteria	0.00–7.17	1.14	4.84	1.94	0.17	3.887 × 10^−3^	9.727 × 10^−2^	12/20
Nitrospiria	0.00–3.54	1.06	0.61	1.68	1.03	2.138 × 10^−1^	5.697 × 10^−1^	17/20
Planctomycetes	0.07–3.46	1.03	0.85	0.87	1.1	8.135 × 10^−1^	9.151 × 10^−1^	20/20
p__Chloroflexota; c__Gitt-GS-136	0.00–4.17	0.87	1.91	1.49	0.51	4.119 × 10^−1^	7.012 × 10^−1^	16/20
p__Gemmatimonadota; c__S0134_terrestrial_group	0.00–3.58	0.78	0.42	1.73	0.66	2.279 × 10^−1^	5.697 × 10^−1^	15/20
Dehalococcoidia	0.00–3.05	0.72	0.64	1.41	0.59	5.725 × 10^−1^	7.361 × 10^−1^	15/20
Parcubacteria	0.00–3.30	0.7	0.07	0.87	0.79	3.475 × 10^−1^	7.012 × 10^−1^	16/20
Cyanobacteriia	0.00–7.14	0.69	2.38	1.87	0.08	9.248 × 10^−1^	9.248 × 10^−1^	7/20
p__Gemmatimonadota; c__PAUC43f_marine_benthic_group	0.00–4.11	0.69	0	0.64	0.85	1.866 × 10^−1^	5.697 × 10^−1^	11/20
p__Gemmatimonadota; c__BD2-11_terrestrial_group	0.00–4.21	0.65	0	0.48	0.82	1.847 × 10^−1^	5.697 × 10^−1^	11/20
Acidobacteriae	0.00–1.94	0.61	0.7	1	0.5	2.283 × 10^−1^	5.697 × 10^−1^	16/20
Desulfuromonadia	0.00–2.53	0.58	0.34	0.67	0.61	6.067 × 10^−1^	7.446 × 10^−1^	15/20
Chloroflexia	0.00–3.14	0.58	1.05	1.22	0.34	5.481 × 10^−1^	7.361 × 10^−1^	12/20

## Data Availability

All data from this project is publicly available (NCBI, BioSample Ac#: SAMN54923591-SAMN54923610).

## Supplementary Materials

The following supporting information can be downloaded at: https://www.mdpi.com/article/10.3390/microorganisms14061337/s1, Table S1: Long-term climatic parameters of soil sampling sites in four regions; Table S2: Classification of soil samples based on physicochemical properties; Table S3: Phylum-level relative abundance (%) of microbial communities in individual soil samples; Table S4: Summary statistics of dominant and subdominant microbial phyla across soil salinity classes; Table S5: Class-level relative abundance (%) of microbial communities in individual soil samples; Table S6: Summary statistics of dominant and subdominant microbial class across soil salinity classes.

## References

[1] Shokri N., Hassani A., Sahimi M. (2024). Multi-scale soil salinization dynamics from global to pore scale: A review. Rev. Geophys..

[2] Cuevas J., Daliakopoulos I.N., del Moral F., Hueso J.J., Tsanis I.K. (2019). A review of soil-improving cropping systems for soil salinization. Agronomy.

[3] Singh A. (2022). Soil salinity: A global threat to sustainable development. Soil Use Manag..

[4] FAO (2024). Global Status of Salt-Affected Soils—Main Report.

[5] Mwesige F.F. (2025). The extent and distribution of salt-affected soils in sub-Saharan Africa from 1970 to the present: A review of the current state of knowledge. Front. Soil Sci..

[6] Negacz K., Malek Ž., de Vos A., Vellinga P. (2022). Saline soils worldwide: Identifying the most promising areas for saline agriculture. J. Arid. Environ..

[7] Hassani A., Azapagic A., Shokri N. (2021). Global predictions of primary soil salinization under changing climate in the 21st century. Nat. Commun..

[8] Wang L., Hu P., Zheng H., Bai J., Liu Y., Hellwich O., Liu T., Chen X., Bao A. (2025). An Automated Framework for Interaction Analysis of Driving Factors on Soil Salinization in Central Asia and Western China. Remote Sens..

[9] Dong X., Ding J., Ge X. (2024). Future changes in soil salinization across Central Asia under CMIP6 forcing scenarios. Land Degrad. Dev..

[10] Zheleznova I., Gushchina D., Meiramov Z., Olchev A. (2022). Temporal and spatial variability of dryness conditions in Kazakhstan during 1979–2021 based on reanalysis data. Climate.

[11] Asanbaev I.K., Faizov K.S. (2007). Pochvovedenie s Osnovami Ekologii i Geografii Pochv (Soil Science with Basic Soil Ecology and Geography).

[12] Issanova G., Abuduwaili J., Mamutov Z.U., Kaldybaev A., Saparov G., Bazarbaeva T. (2017). Saline soils and identification of salt accumulation provinces in Kazakhstan. Arid Ecosyst..

[13] Chen Q., Song Y., An Y., Lu Y., Zhong G. (2024). Soil microorganisms: Their role in enhancing crop nutrition and health. Diversity.

[14] Falkowski P.G., Fenchel T., Delong E.F. (2008). The microbial engines that drive Earth’s biogeochemical cycles. Science.

[15] Fierer N. (2017). Embracing the unknown: Disentangling the complexities of the soil microbiome. Nat. Rev. Microbiol..

[16] Kumawat C., Kumar A., Parshad J., Sharma S.S., Patra A., Dogra P., Yadav G.K., Dadhich S.K., Verma R., Kumawat G.L. (2022). Microbial diversity and adaptation under salt-affected soils: A review. Sustainability.

[17] Zhang G., Bai J., Zhai Y., Jia J., Zhao Q., Wang W., Hu X. (2024). Microbial diversity and functions in saline soils: A review from a biogeochemical perspective. J. Adv. Res..

[18] Durasov A.M., Tazabekov T.T. (1981). Pochvy Kazakhstana [Soils of Kazakhstan].

[19] Delgado-Baquerizo M., Oliverio A.M., Brewer T.E., Benavent-González A., Eldridge D.J., Bardgett R.D., Maestre F.T., Singh B.K., Fierer N. (2018). A global atlas of the dominant bacteria found in soil. Science.

[20] Fierer N., Jackson R.B. (2006). The diversity and biogeography of soil bacterial communities. Proc. Natl. Acad. Sci. USA.

[21] Lauber C.L., Hamady M., Knight R., Fierer N. (2009). Pyrosequencing-based assessment of soil pH as a predictor of soil bacterial community structure at the continental scale. Appl. Environ. Microbiol..

[22] Bahram M., Hildebrand F., Forslund S.K., Anderson J.L., Soudzilovskaia N.A., Bodegom P.M., Bengtsson-Palme J., Anslan S., Coelho L.P., Harend H. (2018). Structure and function of the global topsoil microbiome. Nature.

[23] Thompson L.R., Sanders J.G., McDonald D., Amir A., Ladau J., Locey K.J., Prill R.J., Tripathi A., Gibbons S.M., Ackermann G. (2017). A communal catalogue reveals Earth’s multiscale microbial diversity. Nature.

[24] Bardgett R.D., Van Der Putten W.H. (2014). Belowground biodiversity and ecosystem functioning. Nature.

[25] Philippot L., Raaijmakers J.M., Lemanceau P., Van Der Putten W.H. (2013). Going back to the roots: The microbial ecology of the rhizosphere. Nat. Rev. Microbiol..

[26] ISO (1995). Soil Quality: Determination of Organic and Total Carbon After Dry Combustion (Elementary Analysis).

[27] Nelson D.W., Sommers L.E. (1982). Total carbon, organic carbon, and organic matter. Methods of Soil Analysis: Part 2 Chemical and Microbiological Properties.

[28] Richards L.A. (1954). Diagnosis and Improvement of Saline and Alkali Soils.

[29] Olsen S.R. (1954). Estimation of Available Phosphorus in Soils by Extraction with Sodium Bicarbonate.

[30] Olsen S.R., Sommers L.E., Page A.L. (1982). Phosphorus. Methods of Soil Analysis. Part 2: Chemical and Microbiological Properties.

[31] (2023). Soil Quality—Determination of Exchangeable Cations in Soil and Related Materials Using Extractants.

[32] (2021). Soil, Treated Biowaste and Sludge—Determination of pH.

[33] Baird R., Eaton A.D., Rice E.W., Bridgewater L., Association A.P.H., Association A.W.W., Federation W.E. (2017). Standard Methods for the Examination of Water and Wastewater.

[34] Biver S., Vandenbol M. (2013). Characterization of three new carboxylic ester hydrolases isolated by functional screening of a forest soil metagenomic library. J. Ind. Microbiol. Biotechnol..

[35] Amplicon P., Clean-Up P., Index P. (2013). 16S Metagenomic Sequencing Library Preparation.

[36] Klindworth A., Pruesse E., Schweer T., Peplies J., Quast C., Horn M., Glöckner F.O. (2013). Evaluation of general 16S ribosomal RNA gene PCR primers for classical and next-generation sequencing-based diversity studies. Nucleic Acids Res..

[37] Usyk M., Zolnik C.P., Patel H., Levi M.H., Burk R.D. (2017). Novel ITS1 fungal primers for characterization of the mycobiome. MSphere.

[38] Joshi N., Fass J. (2011). Sickle: A Sliding-Window, Adaptive, Quality-Based Trimming Tool for FastQ Files, Version 1.33.

[39] Bolyen E., Rideout J.R., Dillon M.R., Bokulich N.A., Abnet C.C., Al-Ghalith G.A., Alexander H., Alm E.J., Arumugam M., Asnicar F. (2019). Reproducible, interactive, scalable and extensible microbiome data science using QIIME 2. Nat. Biotechnol..

[40] Callahan B.J., McMurdie P.J., Rosen M.J., Han A.W., Johnson A.J.A., Holmes S.P. (2016). DADA2: High-resolution sample inference from Illumina amplicon data. Nat. Methods.

[41] Bokulich N.A., Kaehler B.D., Rideout J.R., Dillon M., Bolyen E., Knight R., Huttley G.A., Gregory Caporaso J. (2018). Optimizing taxonomic classification of marker-gene amplicon sequences with QIIME 2’s q2-feature-classifier plugin. Microbiome.

[42] Quast C., Pruesse E., Yilmaz P., Gerken J., Schweer T., Yarza P., Peplies J., Glöckner F.O. (2012). The SILVA ribosomal RNA gene database project: Improved data processing and web-based tools. Nucleic Acids Res..

[43] Abarenkov K., Nilsson R.H., Larsson K.-H., Taylor A.F., May T.W., Frøslev T.G., Pawlowska J., Lindahl B., Põldmaa K., Truong C. (2024). The UNITE database for molecular identification and taxonomic communication of fungi and other eukaryotes: Sequences, taxa and classifications reconsidered. Nucleic Acids Res..

[44] Martin M. (2011). Cutadapt removes adapter sequences from high-throughput sequencing reads. EMBnet J..

[45] Abarenkov K., Zirk A., Piirmann T., Pöhönen R., Ivanov F., Nilsson R.H., Kõljalg U. (2025). UNITE QIIME Release for Fungi.

[46] Rognes T., Flouri T., Nichols B., Quince C., Mahé F. (2016). VSEARCH: A versatile open source tool for metagenomics. PeerJ.

[47] R Core Team (2014). R: A Language and Environment for Statistical Computing.

[48] Wickham H., Averick M., Bryan J., Chang W., McGowan L.D.A., François R., Grolemund G., Hayes A., Henry L., Hester J. (2019). Welcome to the Tidyverse. J. Open Source Softw..

[49] Oksanen J., Simpson G.L., Blanchet F.G., Kindt R., Legendre P., Minchin P.R., O’Hara R.B., Solymos P., Stevens M.H.H., Szoecs E. (2022). vegan: Community Ecology Package; R Package Version 2.6-4. https://CRAN.R-project.org/package=vegan.

[50] Wickham H. (2016). Data analysis. ggplot2: Elegant Graphics for Data Analysis.

[51] Chao A. (1984). Nonparametric estimation of the number of classes in a population. Scand. J. Stat..

[52] Shannon C.E. (1948). A mathematical theory of communication. Bell Syst. Tech. J..

[53] Good I.J. (1953). The population frequencies of species and the estimation of population parameters. Biometrika.

[54] Whittaker R.H. (1972). Evolution and measurement of species diversity. Taxon.

[55] Clarke K.R. (1993). Non-parametric multivariate analyses of changes in community structure. Aust. J. Ecol..

[56] Ramette A. (2007). Multivariate analyses in microbial ecology. FEMS Microbiol. Ecol..

[57] Kolde R. (2019). pheatmap: Pretty Heatmaps; R Package Version 1.0.12. https://CRAN.R-project.org/package=pheatmap.

[58] Paliy O., Shankar V. (2016). Application of multivariate statistical techniques in microbial ecology. Mol. Ecol..

[59] FAO, ITPS (2021). Global assessment of salt-affected soils. Food and Agriculture Organization of the United Nations and Intergovernmental Technical Panel on Soils.

[60] Rath K.M., Fierer N., Murphy D.V., Rousk J. (2019). Linking bacterial community composition to soil salinity along environmental gradients. ISME J..

[61] Kaminsky L.M., Trexler R.V., Malik R.J., Hockett K.L., Bell T.H. (2019). The inherent conflicts in developing soil microbial inoculants. Trends Biotechnol..

[62] O’Callaghan M., Ballard R.A., Wright D. (2022). Soil microbial inoculants for sustainable agriculture: Limitations and opportunities. Soil Use Manag..

[63] Parnell J.J., Berka R., Young H.A., Sturino J.M., Kang Y., Barnhart D.M., DiLeo M.V. (2016). From the lab to the farm: An industrial perspective of plant beneficial microorganisms. Front. Plant Sci..

[64] Singh U.B., Malviya D., Singh S., Singh P., Ghatak A., Imran M., Rai J.P., Singh R.K., Manna M.C., Sharma A.K. (2021). Salt-tolerant compatible microbial inoculants modulate physio-biochemical responses enhance plant growth, Zn biofortification and yield of wheat grown in saline-sodic soil. Int. J. Environ. Res. Public Health.

[65] Chen Q., Hu H., He Z., Cui L., Zhu Y., He J. (2021). Potential of indigenous crop microbiomes for sustainable agriculture. Nat. Food.

[66] Del-Canto A., Sanz-Saez Á., Sillero-Martínez A., Mintegi E., Lacuesta M. (2023). Selected indigenous drought tolerant rhizobium strains as promising biostimulants for common bean in Northern Spain. Front. Plant Sci..

[67] Dwivedi S.L., Vetukuri R.R., Kelbessa B.G., Gepts P., Heslop-Harrison P., Araujo A.S., Sharma S., Ortiz R. (2025). Exploitation of rhizosphere microbiome biodiversity in plant breeding. Trends Plant Sci..

[68] Feng G., Wu Y., Yang C., Zhang Q., Wang S., Dong M., Wang Y., Qi H., Guo L. (2024). Effects of coastal saline-alkali soil on rhizosphere microbial community and crop yield of cotton at different growth stages. Front. Microbiol..

[69] Li X., Wang A., Wan W., Luo X., Zheng L., He G., Huang D., Chen W., Huang Q. (2021). High salinity inhibits soil bacterial community mediating nitrogen cycling. Appl. Environ. Microbiol..

[70] Zhang X., Qi L., Li W., Hu B.X., Dai Z. (2021). Bacterial community variations with salinity in the saltwater-intruded estuarine aquifer. Sci. Total Environ..

[71] Marinari S., Carbone S., Antisari L.V., Grego S., Vianello G. (2012). Microbial activity and functional diversity in Psamment soils in a forested coastal dune-swale system. Geoderma.

[72] Kamble P.N., Gaikwad V.B., Kuchekar S.R., Bååth E. (2014). Microbial growth, biomass, community structure and nutrient limitation in high pH and salinity soils from Pravaranagar (India). Eur. J. Soil Biol..

[73] Rousk J., Elyaagubi F.K., Jones D.L., Godbold D.L. (2011). Bacterial salt tolerance is unrelated to soil salinity across an arid agroecosystem salinity gradient. Soil Biol. Biochem..

[74] Zhao Q., Bai J., Gao Y., Zhao H., Zhang G., Cui B. (2020). Shifts in the soil bacterial community along a salinity gradient in the Yellow River Delta. Land Degrad. Dev..

[75] Guellout M., Guellout Z., Belhadj H., Guellout A., Bravo A.G., Jaouani A. (2026). Metagenomic Profiling Reveals the Role of Soil Chemistry–Climate Interactions in Shaping the Bacterial Communities and Functional Repertories of Algerian Drylands. Eng.

[76] Gunde-Cimerman N., Plemenitaš A., Oren A. (2018). Strategies of adaptation of microorganisms of the three domains of life to high salt concentrations. FEMS Microbiol. Rev..

[77] Oren A. (2008). Microbial life at high salt concentrations: Phylogenetic and metabolic diversity. Saline Syst..

[78] Weinisch L., Kühner S., Roth R., Grimm M., Roth T., Netz D.J., Pierik A.J., Filker S. (2018). Identification of osmoadaptive strategies in the halophile, heterotrophic ciliate Schmidingerothrix salinarum. PLoS Biol..

[79] Canfora L., Bacci G., Pinzari F., Lo Papa G., Dazzi C., Benedetti A. (2014). Salinity and bacterial diversity: To what extent does the concentration of salt affect the bacterial community in a saline soil?. PLoS ONE.

[80] Zhang J., Yan Y., Jiang D., Huangfu S., Ma H., Tian L., Huang L. (2025). The Microbial Functional Communities of Mollisol and Saline–Sodic Paddy Soils at Rice Heading and Harvest Stages in Northeast China. Agriculture.

[81] Zhang Y., Wang H., Zhang X., Feng Z., Liu J., Wang Y., Shang S., Xu J., Liu T., Liu L. (2024). Effects of salt stress on the rhizosphere soil microbial communities of *Suaeda salsa* (L.) Pall. in the Yellow River Delta. Ecol. Evol..

[82] Yang C., Li K., Lv D., Jiang S., Sun J., Lin H., Sun J. (2021). Inconsistent response of bacterial phyla diversity and abundance to soil salinity in a Chinese delta. Sci. Rep..

[83] Zhao X.-Y., Gao J.-L., Yu X.-F., Borjigin Q.-G., Qu J., Zhang B.-Z., Zhang S.-N., Li Q., Guo J.-A., Li D.-B. (2024). Evaluation of the microbial community in various saline alkaline-soils driven by soil factors of the Hetao Plain, Inner Mongolia. Sci. Rep..

[84] Cai S., Xu S., Zhang D., Liang Y., Zhu H. (2025). Vegetation-Driven Changes in Soil Salinity Ions and Microbial Communities Across Tidal Flat Reclamation. Microorganisms.

[85] Cui Y., Ning Z., Li M., Qin X., Yue X., Chen X., Zhu C., Sun H., Huang Y. (2025). Microbial network-driven remediation of saline-alkali soils by salt-tolerant plants. Front. Microbiol..

[86] Liu D., Gao P., Niu J., Qu Z., Guo S., Ding C., Lou Y., Yang Q., Wang H., Yang Z. (2024). Assessment of microbial diversity in various saline soils driven by salt content. Pedobiologia.

[87] Bai S., Shang K., Zeng S., Huang Z., Han Z. (2024). Genome analysis of Salinimicrobium sp. 3283s, a deep-sea bacterium isolated from the sediments of South China Sea, China. Mar. Genom..

[88] Chen S., Hou H., Zhang X., Gao Z., Wang H., Yuan Y., Feng B. (2025). Relationship between nutrient accumulation in broomcorn millet (*Panicum miliaceum* L.) and microbial community under different salinity soils. Plant Soil.

[89] Hui R., Tan H. (2024). Divergence of nutrients, salt accumulation, bacterial community structure and diversity in soil after 8 years of flood irrigation with surface water and groundwater. BMC Microbiol..

[90] Nazarov A., Nechaeva Y.I., Korsakova E., Pyankova A., Plotnikova E. (2024). Soil bacterial communities in the affected zone of salt dump (Solikamsk, Perm krai). Eurasian Soil Sci..

[91] Ayayee P.A., Custer G.F., Tronstad L.M., van Diepen L.T. (2024). Unveiling salinity-driven shifts in microbial community composition across compartments of naturally saline inland streams. Hydrobiologia.

[92] Murphy C.L., Biggerstaff J., Eichhorn A., Ewing E., Shahan R., Soriano D., Stewart S., VanMol K., Walker R., Walters P. (2021). Genomic characterization of three novel Desulfobacterota classes expand the metabolic and phylogenetic diversity of the phylum. Environ. Microbiol..

[93] Xu J., Chen L., Zhou T., Zhang C., Zhang J., Zhao B. (2025). Salinity-driven differentiation of bacterial and fungal communities in coastal wetlands: Contrasting assembly processes and spatial dynamics. Environ. Res..

[94] Duran R., Cary J.W., Calvo A.M. (2010). Role of the osmotic stress regulatory pathway in morphogenesis and secondary metabolism in filamentous fungi. Toxins.

[95] Shree A., Pal S., Verma P.K. (2024). Structural diversification of fungal cell wall in response to the stress signaling and remodeling during fungal pathogenesis. Physiol. Mol. Biol. Plants.

[96] Treseder K.K., Lennon J.T. (2015). Fungal traits that drive ecosystem dynamics on land. Microbiol. Mol. Biol. Rev..

[97] Egidi E., Delgado-Baquerizo M., Plett J.M., Wang J., Eldridge D.J., Bardgett R.D., Maestre F.T., Singh B.K. (2019). A few Ascomycota taxa dominate soil fungal communities worldwide. Nat. Commun..

[98] Zhang S., Cao Z., Liu S., Hao Z., Zhang X., Sun G., Ge Y., Zhang L., Chen B. (2025). Soil fungal diversity, community structure, and network stability in the southwestern Tibetan Plateau. J. Fungi.

[99] Maestre F.T., Eldridge D.J., Soliveres S., Kéfi S., Delgado-Baquerizo M., Bowker M.A., García-Palacios P., Gaitán J., Gallardo A., Lázaro R. (2016). Structure and functioning of dryland ecosystems in a changing world. Annu. Rev. Ecol. Evol. Syst..

